# Association of *TaD14-4D*, a Gene Involved in Strigolactone Signaling, with Yield Contributing Traits in Wheat

**DOI:** 10.3390/ijms22073748

**Published:** 2021-04-03

**Authors:** Ruifang Liu, Jian Hou, Huifang Li, Ping Xu, Zhengbin Zhang, Xueyong Zhang

**Affiliations:** 1 Key Laboratory of Agricultural Water Resources, Hebei Laboratory of Agricultural Water-Saving, Center for Agricultural Resources Research, Institute of Genetics and Developmental Biology, Chinese Academy of Sciences, Shijiazhuang 050022, China; lrf0720@163.com (R.L.); xuping@sjziam.ac.cn (P.X.); 2 College of Advanced Agricultural Sciences, University of Chinese Academy of Sciences, Beijing 100049, China; 3 Innovation Academy for Seed Design, Chinese Academy of Sciences, Beijing 100101, China; 4 Key Laboratory of Crop Gene Resources and Germplasm Enhancement, Institute of Crop Science, Chinese Academy of Agricultural Sciences, Beijing 100081, China; houjian@caas.cn (J.H.); lihuifang0213@163.com (H.L.)

**Keywords:** *TaD14*, strigolactone signaling, haplotype, effective tillering number, thousand kernel weight, molecular marker, wheat

## Abstract

Tillering is a crucial agronomic trait of wheat; it determines yield and plant architecture. Strigolactones (SLs) have been reported to inhibit plant branching. D14, a receptor of SLs, has been described to affect tillering in rice, yet it has seldomly been studied in wheat. In this study, three *TaD14* homoeologous genes, *TaD14-4A*, *TaD14-4B*, and *TaD14-4D*, were identified. *TaD14-4A*, *TaD14-4B*, and *TaD14-4D* were constitutively expressed, and *TaD14-4D* had a higher expression level in most tissues. TaD14 proteins were localized in both cytoplasm and nucleus. An SNP and a 22 bp insertion/deletion (Indel) at the exon regions of *TaD14-4D* were detected, forming three haplotypes, namely *4D-HapI*, *4D-HapII*, and *4D-HapIII*. Due to the frameshift mutation in the coding region of *4D-HapII*, the interaction of 4D-HapII with TaMAX2 and TaD53 was blocked, which led to the blocking of SL signal transduction. Based on the two variation sites, two molecular markers, namely *dCAPS-250* and *Indel-747*, were developed. Association analysis suggested that haplotypes of *TaD14-4D* were associated with effective tillering number (ETN) and thousand kernel weight (TKW) simultaneously in four environments. The favorable haplotype *4D-HapIII* underwent positive selection in global wheat breeding. This study provides insights into understanding the function of natural variations of *TaD14-4D* and develops two useful molecular markers for wheat breeding.

## 1. Introduction

Plant architecture, an important index determined in great part by shoot branching (tillering in crops), is one of the key factors of the yield component [1]. Shoot branching is under integrated regulation by hormonal, developmental, and environmental factors [2,3]. Strigolactones (SLs) are a group of carotenoid-derived plant hormones, which were demonstrated for the first time in 2008 as regulators that can inhibit the outgrowth of axillary buds, whose function is highly conserved in both monocots and dicots [4,5]. Other functions have been proposed for SLs in the regulation of plant growth and development, including accelerating leaf senescence [6,7], promoting secondary growth of stems [8], regulating plant root development [9], mediating plant tolerance to nutrient deficiency [10], and enhancing plant response to drought and high salt stress [11,12].

The biosynthesis of SLs begins from β-carotene. *D27/AtD27* encodes a β-carotene isomerase, which converts all-trans-β-carotene to 9-cis-β-carotene in the initial steps [13,14,15]. Then, the catalysis of carotenoid cleavage dioxygenase 7 (CCD7) and CCD8 (encoded by *HTD1/D17/MAX3* [16,17,18] and *D10/MAX4* [19] in rice (*Oryza sativa*) and Arabidopsis (*Arabidopsis thaliana*), respectively) produces carlactone (CL) [13], the last common precursor for all SLs [20]. Recent studies have shown that MAX1 and other enzymes can catalyze the biosynthesis of both canonical and noncanonical SLs from CL, whereas this process is different in rice and Arabidopsis [21,22,23].

Studies on a series of SL-insensitive mutants indicated that the SLs signaling mechanism involves targeted degradation of hormone-activated proteins, similar to auxin, jasmonic acid (JA), and gibberellic acid (GA) [20,24]. The perception and signal transduction of SLs in rice and Arabidopsis are coordinated by three highly conserved components [25]: DWARF14 (D14)/AtD14 [26,27], D3/MAX2 [28,29,30], and D53/SMXL6/7/8 [3,31,32]. D14, an α/β-hydrolase protein with a strictly conserved Ser–His–Asp catalytic triad, has been identified as an SL receptor [33,34,35]. Compared with other plant hormone receptors, D14 is unique in that it is a novel type of hormone receptor with dual functions of enzyme and receptor [36]. *D3/MAX2* encodes an F-box protein with a leucine-rich repeat (LRR) sequence, which is involved in the formation of the SCF protein complex [25,28]. In 2013, there was a significant breakthrough in the study of SL signaling transduction. D53, a repressor of SL signaling, was identified as a substrate of the SCF^D3^ ubiquitination complex [3,31].

Wheat is one of the most important staple crops in the world. It is estimated that by 2050, wheat production needs to be increased by 70% from the current level in order to meet the needs of the growing world population [37]. Tillering is a crucial agronomic trait of wheat; it determines yield and plant architecture [38]. The proper number of tillers is of great significance to wheat production. The functions of SLs as plant hormones mainly affect plant branches [4,5]. However, to date, few studies on the genes involved in SL biosynthesis and signal transduction have been reported in wheat, and the roles of these genes in influencing wheat tillering and other agronomic traits remain unclear.

Association analysis based on gene polymorphism has been proved to be an effective method to reveal the relationship between genes and traits and has been widely performed in many plant species, such as for *OsLG3b* in rice [39], *ZmVPP1* in maize [40], and *TaDA1* [41] and *TaBT1* [42] in wheat. A series of key haplotypes that can be distinguished by effective molecular markers and which are associated with important traits have been identified using this method [43]. Moreover, the use of marker-assisted selection (MAS) to accumulate favorable alleles or haplotypes is considered a potential way to accelerate the process of wheat breeding [44]. Hence, the discovery of excellent gene allelic variations and the development of molecular markers have practical value for the breeding and genetic improvement of wheat.

In this study, the major objectives were to (1) isolate and characterize three homeologs of *TaD14* from wheat, (2) explore expression patterns of *TaD14* genes in various tissues and periods, (3) identify sequence diversity and elucidate the possible molecular mechanism of a natural mutation of *TaD14-4D*, (4) develop functional markers to distinguish haplotypes and associate them with agronomic traits, and (5) evaluate the value of the newly developed molecular markers and assess whether different haplotypes were selected by analyzing the geographic distribution and frequency of favorable allelic variation. This study aimed to identify potentially important genes and provide a valuable functional marker for molecular marker-assisted wheat breeding.

## 2. Results

### 2.1. Identification and Structural Analysis of TaD14 Genes

**Figure 1:**
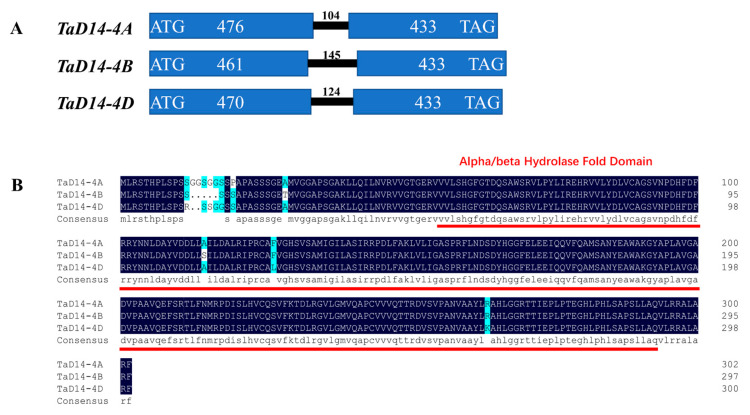
Structures of *TaD14* homoeologous genes and amino acid sequence alignment. (**A**) Gene structures of *TaD14-4A*, *TaD14-4B*, and *TaD14-4D*. The blue rectangles represent exons, the solid black line between exons represents introns, and the number above represents the number of bases. (**B**) The amino acid sequence alignment of TaD14 proteins in Chinese Spring. The predicted α/β-hydrolase fold domain is indicated by the red line.

*Dwarf 14* (*D14*) encodes an α/β-fold hydrolase superfamily protein. Loss of function mutants exhibit dwarf and high tillering phenotype in rice [26]. To investigate the function of *D14* in wheat, the coding sequence (CDS) of *OsD14* (Os03g0203200) was used as a query to search in the Chinese Spring RefSeq v1.0 genome database [45]. Three *D14* homeologs located in chromosomes 4AS (TraesCS4A02G046700), 4BL (TraesCS4B02G258200), and 4DL (TraesCS4D02G258000) were obtained and named *TaD14-4A*, *TaD14-4B*, and *TaD14-4D*. Primers were designed based on specific regions for each sequence (Table S1) to clone the genomic sequence and CDS of *TaD14* genes using genomic DNA and cDNA samples of Chinese Spring. All three homoeologous genes of *TaD14* were composed of two exons and one intron (Figure 1A). The genomic sequence lengths of *TaD14-4A*, *TaD14-4B*, and *TaD14-4D* were 1013, 1039, and 1027 bp, respectively, encoding putative products of 302, 297, and 300 amino acids, respectively (Figure S1). In addition, these proteins had a high identity of 98.01% and all contained an α/β-hydrolase fold domain (Figure 1B), indicating that they could perform a similar function in wheat.

**Figure 2:**
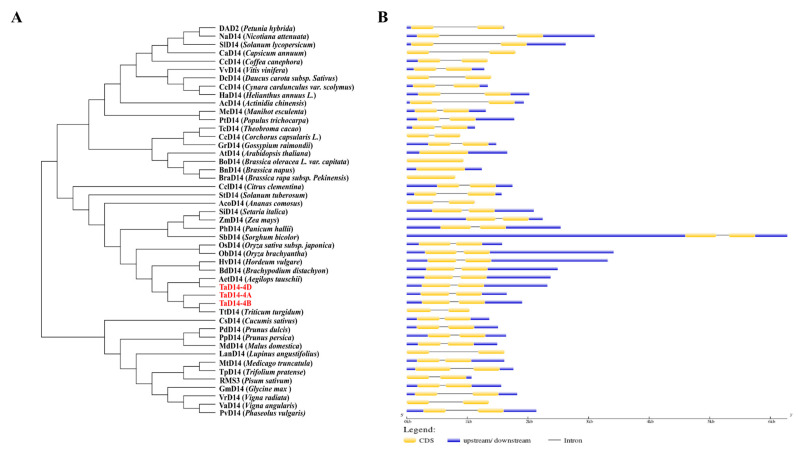
Phylogenetic relationships of D14 proteins in different species and the corresponding *D14* gene structures. (**A**) Phylogenetic tree constructed from the D14 protein sequences of 47 different species using the neighbor-joining method with a bootstrap value of 1000 by MEGA7.0. Three homoeologous proteins of TaD14 are marked in red. (**B**) Gene structures of *D14.* The yellow boxes indicate coding sequences, the blue boxes represent the untranslated regions, and the black lines represent introns. The length of coding sequence (CDS) and introns can be estimated using the scale at the bottom.

The phylogenetic analysis revealed that the TaD14 protein had the closest homology relationship with D14 of *Triticum turgidum*, *Aegilops tauschii*, *Hordeum vulgare*, *Brachypodium distachyon*, and *Oryza sativa* subsp. *Japonica* (Figure 2A). Interestingly, except for four cruciferous plants, the *D14* genes of the other species all contained two exons and one intron (Figure 2B), indicating that they were highly conserved in both dicots and monocots during the evolutionary process.

### 2.2. Expression Patterns of TaD14 Genes and Subcellular Localization of TaD14 Proteins

**Figure 3:**
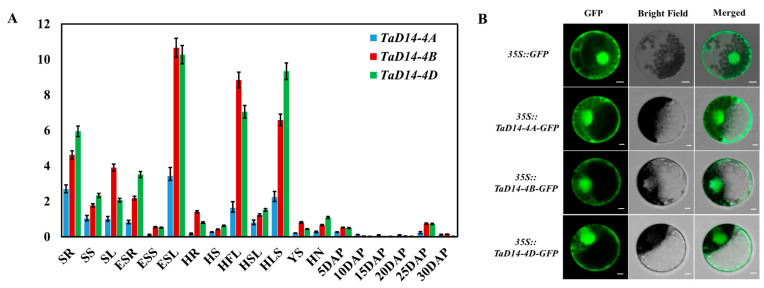
Expression analyses of *TaD14* genes and subcellular localization of TaD14 proteins. (**A**) Tissue-specific expression pattern of *TaD14* genes in different tissues and growth periods of Chinese Spring. SR, seedling roots; SS, seedling stems; SL, seedling leaves; ESR, roots at elongation stage; ESS, stems at elongation stage; ESL, leaves at elongation stage; HR, roots at heading stage; HS, stems at heading stage; HFL, flag leaves at heading stage; HSL, top second leaves at heading stage; HLS, leaf sheaths at heading stage; YS, young spikes; HN, nodes at heading stage; DAP, days after pollination, grains at different developmental stages, namely 5, 10, 15, 20, 25, and 30 DAP. The normalized value of *TaD14* gene expression relative to *TaActin* is derived from the mean ± SD of three technical repeated experiments. (**B**) Subcellular localization of TaD14 proteins in wheat leaf protoplasts. The free *GFP* and *TaD14–GFP* fusions under the control of the cauliflower mosaic virus 35S promoter were transiently expressed in wheat leaf protoplasts. The fluorescence signal of GFP was observed under a confocal laser scanning microscope after transfection for 16 h. Scale bars, 10 μm.

qRT-PCR was performed to explore expression patterns of *TaD14* genes using genome-specific primers (Table S1) so as to clarify the expression of *TaD14* genes in different tissues and growth periods of wheat and to better grasp its dynamic patterns. The expression patterns of *TaD14* genes were similar. They were ubiquitously expressed in all the detected tissues at different stages. The expression levels of *TaD14* genes in leaves were significantly higher than those in other organs, followed by the expression levels in roots. In general, the expression levels of *TaD14-4B* and *TaD14-4D* in all the detected tissues were significantly higher than those of *TaD14-4A* (Figure 3A).

To examine the subcellular localization of TaD14 proteins, we transiently expressed the *p35S–TaD14–GFP* (green fluorescent protein) fusion construct in wheat leaf protoplasts. After overnight incubation (16 h), the protoplasts were analyzed using a confocal microscope. As shown in Figure 3B, TaD14–GFP constructs were localized in both cytoplasm and nucleus, which was consistent with the localization of D14 in Arabidopsis and rice [27,46].

### 2.3. Variations of TaD14-4D among Wheat Accessions

**Figure 4:**
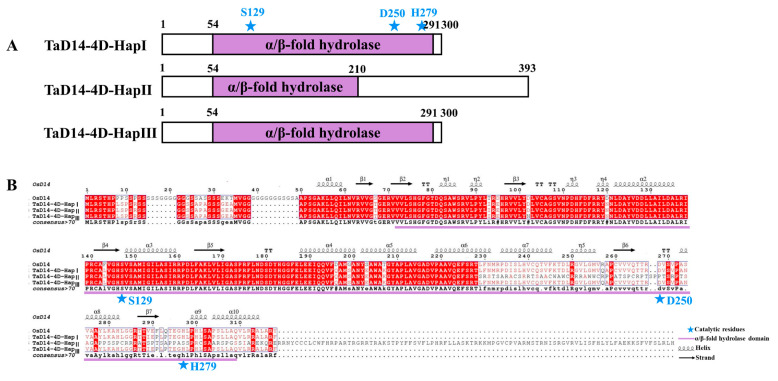
Comparison of protein structures in different haplotypes of TaD14-4D. (**A**) Protein structure in different TaD14-4D haplotypes. The purple boxes represent α/β-hydrolase fold domain and the number above represents the number of amino acids. (**B**) Sequence alignment and structural annotation of TaD14-4D among different haplotypes. Identical and similar residues are highlighted in red and white backgrounds, respectively. The three catalytic residues represented by blue stars, S129, D250, and H279, were deduced from OsD14. The secondary structure of OsD14 was from Nakamura et al. [33]. The purple line indicates α/β-hydrolase fold domain.

Since the identity of TaD14 homoeologs was extremely high and *TaD14-4D* had a higher expression level in most tissues, we speculated that it may have a greater impact on related agronomic traits. To clarify nucleotide natural polymorphisms in *TaD14-4D*, we detected its natural variations in the coding and promoter regions of *TaD14-4D* in 32 wheat accessions (Table S2) with high genetic diversities. No mutation site was present in the promoter region, yet a single nucleotide polymorphism (SNP) in the first exon and a 22 bp insertion/deletion (Indel) in the second exon were detected, which constituted three haplotypes, *4D-HapI*, *4D-HapII*, and *4D-HapIII* (Figure 4).

The SNP at position 250 in the first exon of *4D-HapIII* led to an amino acid change (GAC → AAC, Asp → Asn). The 22 bp Indel in the coding region of *4D-HapII* caused frameshift mutations, which added 93 extra amino acids (Figure 4). The α/β-hydrolase fold domain of 4D-HapII was destroyed, which led to the abnormal catalytic triad composed of the Ser–His–Asp that recognizes and hydrolyzes SLs (Figure 4A). When comparing the amino acid sequences of the three haplotypes (Figure 4B) with the secondary structure of OsD14 [33], the alignment suggests that the mutation sites of *4D-HapII* cause large changes in the secondary structure, which may lead to changes in function.

### 2.4. The SL Signaling Pathway Is Blocked in 4D-HapII

In order to explore the functional defect of D14 in 4D-HapII, subcellular localization of the 4D-HapII was investigated first. The 4D-HapII and TaD14 had the same subcellular localization pattern, both localized in cytoplasm and nucleus (Figures S2 and Figure 3B), which indicates that frameshift mutations did not disturb localization. In rice, OsD14 could perceive SL signal and form a complex with signaling pathway molecules OsD3 and OsD53 to mediate SL signal transduction as a bifunctional protein [3,31]. The subcellular localization patterns of TaMAX2 and TaD53 (ortholog of OsD3 and OsD53 in wheat), two other members of the SL signaling pathway in wheat, were both localized in the nucleus (Figure S2). The subcellular localization of GFP-4D-HapII overlapped with TaMAX2 and TaD53. Therefore, we investigated whether 4D-HapII could interact with them.

**Figure 5:**
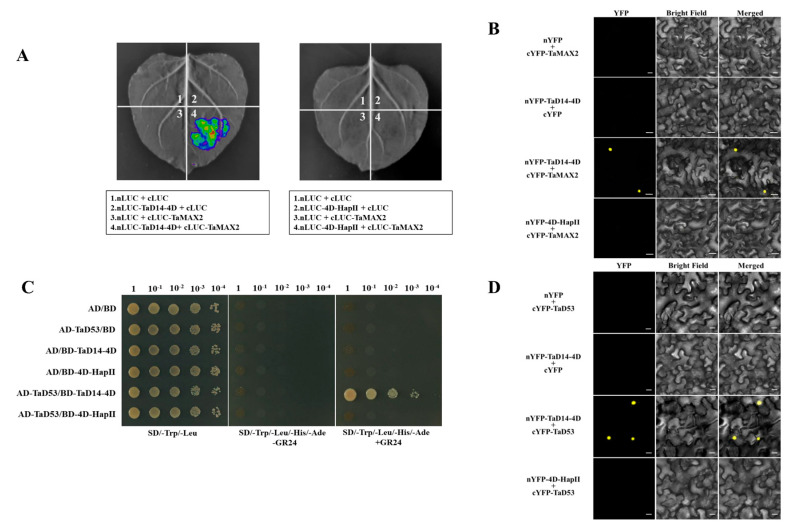
The interactions between TaD14-4D haplotypes and strigolactone pathway members TaMAX2 and TaD53. (**A**) LCI assay showed TaMAX2 interacts with TaD14-4D instead of 4D-HapII. nLUC, N-terminal of LUC; cLUC, C-terminal of LUC. (**B**) BiFC assay showed TaMAX2 interacts with TaD14-4D instead of 4D-HapII. YFP, yellow fluorescent protein. nYFP and cYFP represent the N-terminal and C-terminal of YFP, respectively. BF, bright-field. Scale bars, 20 μm. (**C**) Yeast two-hybrid assay showed TaD53 interacts with TaD14-4D instead of 4D-HapII. AD, activating domain; BD, binding domain; SD, synthetic dropout medium. Yeast transformants were spotted on the control medium (SD/Trp/Leu, SD lacking Trp and Leu) and selective medium (SD/Trp/Leu/His/Ade, SD lacking Trp, Leu, His, and Ade). The yeast transformants were 10-fold serially diluted and spotted onto the selected medium. (**D**) BiFC assay showed TaD53 interacts with TaD14-4D instead of 4D-HapII. Scale bars, 20 μm. Three independent tobacco leaves were used for LUC and YFP signal detection.

To test the interaction of 4D-HapII with TaMAX2, a firefly luciferase complementation imaging (LCI) assay was employed in tobacco (*Nicotiana benthamiana*) leaves. As shown in Figure 5A, TaD14-4D directly interacted with TaMAX2, while 4D-HapII could not interact. We further investigated the interaction using the bimolecular fluorescence complementation (BiFC) assays. For this experiment, TaD14-4D and 4D-HapII were fused with the split amino-terminus of yellow fluorescent protein (YFP) protein and TaMAX2 was fused with the split carboxy-terminus of YFP protein to generate nYFP-TaD14-4D, nYFP-4D-HapII, and cYFP-TaMAX2. Consistently, BiFC assays also demonstrated that TaD14-4D could directly interact with TaMAX2 in the nucleus, while 4D-HapII could not (Figure 5B).

A yeast two-hybrid (Y2H) assay was set up to detect the differences in TaD14-4D and 4D-HapII interaction with TaD53. The results suggested that neither TaD14-4D nor 4D-HapII could physically interact with TaD53 in the absence of GR24, an artificial SL analog (Figure 5C). When the final concentration of 5 μm GR24 was added to the yeast selective medium, TaD14-4D could interact with TaD53 protein in the presence of GR24, but 4D-HapII could not (Figure 5C). Simultaneously, BiFC assays also confirmed that 4D-HapII could not interact with TaD53 (Figure 5D). The above results indicate that the MAX2–D14–D53 protein complex with D14 as the center could not form in 4D-HapII. As a consequence, the ubiquitin ligase TaMAX2 was unable to physically approach the substrate TaD53, resulting in blocked SL signaling transduction.

### 2.5. Molecular Marker Development of TaD14-4D Haplotypes and Association Analysis with Agronomic Traits

**Figure 6:**
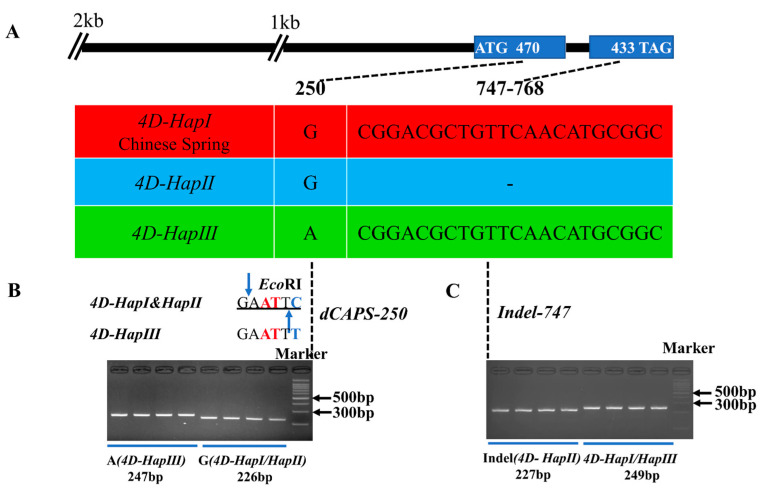
Haplotypes of *TaD14-4D* and the molecular marker development. (**A**) The top panel shows a schematic diagram of the gene and 2 kb promoter structure of *TaD14-4D*. The ATG start codon was designated as position 1 bp. The bottom panel shows the polymorphic sites of *TaD14-4D*. (**B**) A dCAPS marker named *dCAPS-250* was developed based on the 250 bp single-nucleotide polymorphism (G/A). Digestion of the amplified 247 bp fragment with *Eco*RI produced fragments of 226 bp and 21 bp for accessions with the haplotype *4D-HapI*/*II* (G), whereas this fragment was not digested in accessions with the haplotype *4D-HapIII* (A). The blue base represents the reverse complementarity of the mutation site, the red base represents the introduced mismatch bases, the black line represents the base recognized by the restriction enzyme, and the blue arrow represents the restriction enzyme site. (**C**) An Indel marker named *Indel-747* was developed based on a deletion of 22 bp.

Based on the polymorphic sites, two molecular markers were developed to distinguish the three haplotypes, named *dCAPS-250* and *Indel-747*. Based on the SNP(G/A) at base pair 250 (Figure 6A), a derived cleaved amplified polymorphic sequence (dCAPS) marker was developed to distinguish *4D-HapIII* from *4D-HapI* and *4D-HapII* (Figure 6B). The marker contained two mismatches in the downstream primer that produced a recognition site for the restriction enzyme *Eco*RI at *4D-HapI* and *4D-HapII*, but not at *4D-HapIII* (Figure 6B, Table S1). After two-step PCR amplification and enzyme digestion, the amplified fragments of *4D-HapIII* could be separated (Figure 6B). The Indel marker was developed based on a deletion of 22 bp at base pair 747 to discriminate *4D-HapII* (Figure 6C). After two-step amplification, the PCR products could be distinguished by 4% agarose gel (Figure 6C).

**Figure 7:**
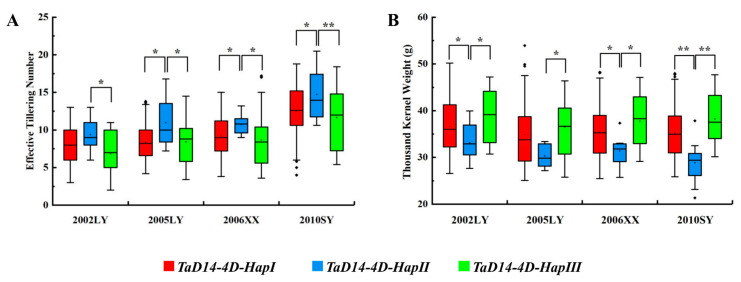
Association analysis of *TaD14-4D* haplotypes with effective tillering number and thousand kernel weight in the mini-core collection (MCC). (**A**,**B**) Association of *TaD14-4D* haplotypes with effective tillering number (**A**) and thousand kernel weight (**B**) in the mini-core collection (MCC) in four different environments; the x-axis represents different environments (Luoyang, 2002; Luoyang, 2005; Xinxiang, 2006; Shunyi, 2010). The asterisks indicate significant differences between haplotypes (Student’s *t*-test, * *p* < 0.05, ** *p* < 0.01).

The association between haplotypes and agronomic traits was analyzed in a mini-core collection (MCC) of 262 wheat accessions [47]. After scanning 262 MCC members by the two molecular markers *dCAPS-250* and *Indel-747*, we associated the three haplotypes with 11 agronomic traits collected from MCC members grown under various environments in different years. According to the results, *TaD14-4D* haplotypes were significantly correlated with effective tillering number (ETN) (Figure 7A, Table 1). The mean ETN of *4D-HapII* was significantly higher than that of *4D-HapI/III* plants by 1.45–2.56 in 2002, 2.07–2.55 in 2005, 1.89–2.47 in 2006, and 2.14–3.08 in 2010 (Figure 7A, Table 1). Significant differences were also detected in thousand kernel weight (TKW) (Figure 7B). Interestingly, the TKW of *4D-HapII* was significantly lower than that of *4D-HapI/III*. These results showed that *4D-HapII* had phenotypes of ETN and TKW similar to those of a series of SL signal transduction mutants *dwarf14* in rice, including *d14*, *d88* [48], *htd2* [49], and *htd4* [50]. Furthermore, accessions with *4D-HapIII* had a lower mean ETN and higher TKW (not reaching a significant level), and *4D-HapI* was an intermediate haplotype. In summary, these results indicate that *4D-HapIII* was the favorable haplotype.

### 2.6. Global Distribution of TaD14-4D Haplotypes

**Figure 8:**
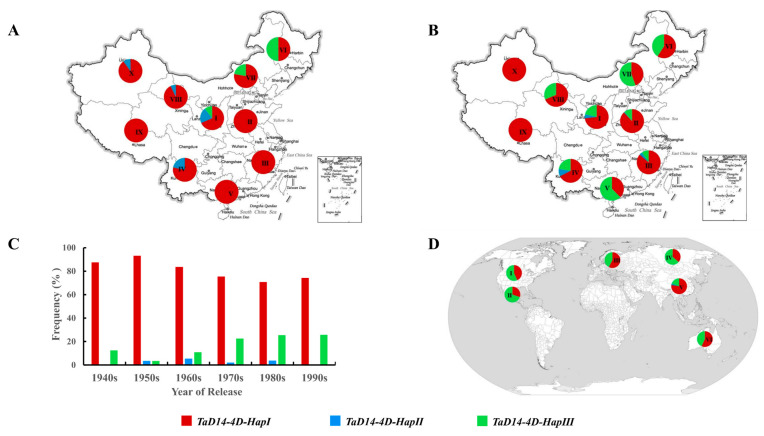
Spatial and temporal distribution of *TaD14-4D* haplotypes. (**A**,**B**) Distribution of *TaD14-4D* haplotypes in ten Chinese ecological regions (I, northern winter wheat region; II, Yellow and Huai River valley winter wheat region; III, low and middle Yangtze River valley winter wheat region; IV, southwestern winter wheat region; V, southern winter wheat region; VI, northeastern spring wheat region; VII, northern spring wheat region; VIII, northwestern spring wheat region; IX, Qinghai–Tibet spring–winter wheat region; X, Xinjiang winter–spring wheat region). (**A**) Representation of 157 landraces from ten Chinese ecological regions. (**B**) Representation of 348 modern cultivars from ten Chinese ecological regions. (**C**) The changes in the frequency of *TaD14-4D* haplotypes in Chinese wheat breeding history (from the 1940s to the 2000s). (**D**) Distribution of *TaD14-4D* haplotypes in major global wheat ecological regions (I, North America; II, CIMMYT; III, Europe; IV, former USSR; V, China; VI, Australia).

To determine the distributions of different haplotypes of *TaD14-4D* and whether they were selected, 157 landraces from the MCC and 348 cultivars from the modern cultivars (MC) covering 10 different wheat production zones of China were genotyped by the two markers *dCAPS-250* and *Indel-747*. From landraces to modern cultivars, the proportions of *4D-HapIII* were higher in seven ecological wheat zones, especially in the major production zones (I–IV) and this was consistent with the aforementioned preferred haplotype *4D-HapIII* (Figure 8A,B). By contrast, the frequency of *4D-HapII* declined during the transition from landraces to modern cultivars. These results showed that the *4D-HapIII* underwent positive selection in the process of wheat breeding in China.

Next, the frequency change of the *TaD14-4D* haplotypes during Chinese wheat breeding since the 1940s was surveyed. The frequencies of favorable *4D-HapIII* haplotype increased from 12.5% to 25.7%, but those of *4D-HapII* were gradually eliminated after 1990 (Figure 8C). The proportions of intermediate *4D-HapI* were at a relatively high level, and the favored *4D-HapIII* had large selection potential.

Finally, we performed genotyping analysis of modern cultivars from Australia, the International Maize and Wheat Improvement Center (CIMMYT), Europe, the former USSR, and North America to assess the global geographic distribution pattern of the *TaD14-4D* haplotypes (Figure 8D). Similar to the selection trend in China, the proportion of *4D-HapIII* was also high in the other five regions, especially in North America, Mexico, and the former USSR. In these regions, the proportion of *4D-HapIII* was higher than that of *4D-HapI*, and the proportion of *4D-HapII* was very low. These results provide strong evidence that *4D-HapIII* underwent positive selection in global wheat breeding.

## 3. Discussion

### 3.1. Functional Conservation of D14 between Wheat and Other Plants

Strigolactones are a new class of plant hormones. SLs, as carotenoid derivatives, are involved in regulating the growth and development process in a variety of plants [51]. Plant branching is an important agronomic trait that affects plant spatial structure and determines crop yield. Therefore, the study of SL hormones is of great significance to the improvement of plant architecture. The previous studies used forward genetics to screen mutants and confirmed that *AtD14* and *D14* inhibit plant branching (tillering), and the *d14* mutant also showed a dwarfed phenotype [26,48,49]. In rice, a series of *D14* allelic mutants had different degrees of decrease in plant height and increase in tillering number, but in general, the degree of change in tillering number was much greater than that of plant height [26,48,49]. For example, *htd4* [50] is a mild phenotypic allelic mutant due to the smaller differences in plant height and tillering number compared with the corresponding wild type. In addition, *d27/Atd27* displayed obviously reduced height and increased tillering (branching) [14,15], but in *TaD27-RNAi*, tillering was increased yet plant height was not significantly changed [52]. In this study, the *TaD14* genes were cloned and identified in common wheat. Through the analysis of the natural variation types of the *TaD14-4D*, three haplotypes were detected (Figure 4 and Figure 6). The haplotypes were significantly associated with effective tillering number (ETN) but not significantly associated with the plant height (PH) (Figure 7A, Table 1). Although the *4D-HapII* haplotype has amino acid changes due to frameshift mutations (Figure 4), we cannot rule out the possibility that mutations are insufficient to affect plant height. This suggests that SL pathway genes may have nuanced functions in wheat.

### 3.2. Natural Variation of 4D-HapII Influences Protein Function

*D14* encodes a member of α/β-hydrolase superfamily [26]. Studies have shown that D14 is a bifunctional protein functioning as both hydrolase and SL receptor [36]. The crystal structures of DAD2, D14, and AtD14 display that they all have the canonical α/β-hydrolase domain formed by the substrate-binding pocket and the Ser–His–Asp catalytic triad which is necessary for hydrolase activity [27,34]. It is reported that a cap that is crucial is formed by four helices surrounding the entrance to the active site pocket [34,53,54]. In our study, a 22 bp Indel was detected in *4D-HapII*, which was a rare mutation, and this haplotype was found to be gradually eliminated during the global breeding process (Figure 6A and Figure 8). Regarding this, there are two possible reasons. Firstly, natural mutations in *4D-HapII* led to missense mutations, and the hydrolase domain of the protein was destroyed, especially the highly conserved Ser–His–Asp catalytic triad domain (Figure 4 and Figure 6A). Secondly, our results show that the D14 cap structure is formed by α4–α7, as seen in Figure 4B. In 4D-HapII, α6 and α7 cannot form correctly, so we speculate that the cap structure, which is important for D14 to recognize and hydrolyze SLs, cannot function normally. Therefore, SLs cannot be hydrolyzed in 4D-HapII, which may lead to a lack of interaction between D14-MAX2 and D14-D53 (Figure 5).

### 3.3. Effective Molecular Markers for Wheat Breeding

In recent years, the use of molecular markers, as an effective means to link phenotypic with genotypic variations, has become a powerful tool for the analysis of genetic variation in the agronomic sector [55,56]. Markers also allow for a more accurate evaluation of genetic resources to identify new and original alleles [44]. Wheat germplasm resources include very rich allelic variations. Thus, designing a functional molecular marker is an important prerequisite for marker-assisted breeding. In this study, we developed two molecular markers, *dCAPS-250* and *Indel-747*, to distinguish haplotypes, and they were associated with ETN and TKW (Figure 6 and Figure 7, Table 1). The *4D-HapIII* was associated with a higher mean TKW, and its proportion was higher in five wheat production regions than in China (Figure 7 and Figure 8D), so the favorable haplotype *4D-HapIII* could be useful for further selection in China. Thus, the newly developed molecular markers can be used for marker-assisted selection breeding in wheat.

## 4. Materials and Methods

### 4.1. Plant Materials and Growing Conditions

The correlation analysis of field agronomic traits and haplotypes was conducted by using a population composed of 262 MCC members [47]. Field agronomic traits included heading date (HD), maturity date (MD), panicle length (PL), spikelet number per spike (SN), grain number per spike (GN), plant height (PH), effective tiller number (ETN), thousand kernel weight (TKW), kernel length (KL), kernel width (KW), and kernel thickness (KT) [57]. Another population of 348 Chinese modern cultivars was used to analyze the temporal and spatial distribution of different haplotypes. A population of 157 Chinese landraces from the MCC was adopted to study the geographic distribution of different haplotypes. These two groups were planted at Luoyang in 2002 and 2005, Xinxiang (only MCC) in Henan Province in 2006, and Shunyi in Beijing in 2010 [58].

A population consisting of 348 Chinese cultivars, 490 North American cultivars, 384 European cultivars, 51 Australian cultivars, 83 Russian cultivars, and 53 CIMMYT cultivars [59] was utilized to investigate the global geographic distribution of *TaD14-4D* haplotypes.

For tissue expression analysis of the *TaD14* homoeologous genes, Chinese Spring cultivated under long-day greenhouse conditions (16 h light/8 h dark, 22 °C, relative humidity 70%, light intensity 150 µmol m^−2^ s^−1^) was used. Tissue samples of roots, stems, and leaves were taken at seedling stage, jointing stage, and heading stage, respectively, and grain samples were taken at different times after flowering. Meanwhile, Chinese Spring was used for genomic cloning and cDNA cloning of *TaD14* homoeologous genes.

### 4.2. Cloning and Characterization of TaD14 Genes

The CDS sequence of the rice *OsD14* (Os03g0203200) gene published in the China Rice Data Center (http://www.ricedata.cn/gene/index.htm (accessed on 3 January 2020)) was used for a BLAST against IWGSC Survey Sequence Assemblies (https://urgi.versailles.inra.fr/blast/blast.php (accessed on 3 January 2020)). Three homoeologous gene sequences with high similarity to *OsD14* sequence were obtained. According to sequence differences between the three homoeologous genes, specific primers were designed by the Primer Premier 5.0 software and NCBI Primer-BLAST (https://www.ncbi.nlm.nih.gov/tools/primerblast/index.cgi?LINK_LOC=BlastHome (accessed on 5 January 2020)). All primers (Table S1) used in this study were synthesized by the BGI Tech (Shenzhen, China).

The cDNA and genomic DNA of Chinese Spring were used as PCR templates. PCR amplification was performed in a total volume of 10 μL, including 50 ng templates, 1 μL forward and reverse primers (10 μM), 0.096 μL dNTPs (25 mM), 2 μL 5× TransStart *FastPfu* Buffer, and 0.2 μL (2.5 U/μL) TransStart *FastPfu* DNA Polymerase (TransGen Biotech, Beijing, China). PCR was performed with the following procedure: denaturing at 95 °C for 3 min; followed by 35 cycles of denaturing at 95 °C for 45 s, annealing at 55–60 °C for 45 s, and 72 °C for extension (1 kb/min); with a final extension at 72 °C for 10 min. The PCR products were separated by electrophoresis in 1.5% agarose gel. The PCR products were purified by an AxyPrep DNA Gel Extraction Kit (Axygen Biosciences, Hangzhou, China), cloned into *pEASY*-Blunt cloning vector (TransGen Biotech, Beijing, China), and then transformed to Trans1-T1 Phage Resistant Chemically Competent Cells (TransGen Biotech, Beijing, China) by the heat shock method.

### 4.3. RNA Extraction and Gene Expression Analysis

Total RNA from different tissues was extracted with an RNAprep Pure Plant Kit (Tiangen, Beijing, China), and the cDNA was synthesized using the FastKing RT Kit (Tiangen, Beijing, China). The quantitative PCR (qRT-PCR) was adopted to analyze *TaD14* gene expression levels with Roche Light Cycler 96 using the SYBR Premix Ex Taq (Takara, Dalian, China), as previously described [57]. The 20 μL reaction system of qRT-PCR was composed of 2 μL of cDNA, 0.4 μL of each primer (2 μM), 0.4 μL of ROX Reference Dye (50×), and 10 μL of 2× SYBR Premix Ex Taq (Takara, Dalian, China). The qRT-PCR results were obtained for two biological replications, and similar results were observed. Gene expression was calculated from three technical replicates using the ^ΔΔ*Ct*^ method with *TaActin* as the endogenous control.

### 4.4. Phylogenetic Analysis

In order to investigate the evolutionary relationship of TaD14 genes, a BLAST search was performed in the UniProt database (https://www.uniprot.org/blast/ (accessed on 24 October 2020)) based on the conserved amino acid sequences of TaD14. According to the comparison results, we downloaded the amino acid sequences of D14 proteins of these species and then constructed a phylogenetic tree of D14s from a complete alignment of 47 D27 protein sequences by the neighbor-joining method with 1000 bootstrap replicates and p-distance substitution model using MEGA 7.0. The numbers and positions of exons and introns of each *D14* of these species were determined by the comparison of the CDS sequences and the corresponding genomic DNA sequences via GSDS2.0 (Gene Structure Display Server) website (http://gsds.cbi.pku.edu.cn/ (accessed on 3 November 2020)).

### 4.5. Subcellular Localization

For subcellular localization, we amplified the full-length CDS of *TaD14*/*4D-HapII*/*TaMAX2*/*TaD53* without the termination codon with corresponding primers containing 5′ *Hin*dIII and 3′ *Bam*HI sites from cDNA by PCR. Then, the purified PCR products were fused with the green fluorescent protein (GFP) in the pJIT163-GFP vector to construct the recombinant vectors. The procedure to obtain wheat leaf protoplasts was according to Yoo et al. (2007) [60]. Four fusion plasmids and free GFP plasmids were transfected into wheat leaf protoplasts for transient expression. The protoplasts were incubated overnight in the dark at 22 °C, and the green fluorescence signal was observed with a confocal microscope (LSM880; Carl Zeiss, Oberkochen, Germany).

### 4.6. Yeast Two-Hybrid Assay (Y2H)

The coding region of *TaD53* was cloned into the Y2H “prey” vector pGADT7, and the coding regions of *TaD14-4D* and *4D-HapII* were cloned into the Y2H “bait” vector pGBKT7. All constructs were confirmed by sequencing and transformed into the yeast strain Y2H Gold, and the transformants were grown on SD/Trp/Leu plates for 3 days at 30 °C. Yeast strains transformed by *TaD53*, *TaD14-4D*, and *4D-HapII* in combination with empty vector pGADT7 and pGBKT7 were used as negative control. The yeast transformants were 10-fold serially diluted and spotted onto the selected medium. The interactions between the two proteins were determined on the control medium LT (SD/Trp/Leu) and selective medium LTHA (SD/Trp/Leu/His/Ade) in the presence or absence of 5 μM GR24 [3]. Plates were incubated at 30 °C for 3 days.

### 4.7. Luciferase Complementation Imaging (LCI) and Bimolecular Fluorescence Complementation (BiFC) Assay

For the LCI assay, the CDSs of *TaD14-4D* and *4D-HapII* were cloned into n-LUC vectors and the CDS of *TaMAX2* was cloned into c-LUC vector to generate the TaD14-4D–n-LUC, 4D-HapII–nLUC, and TaMAX2–c-LUC constructs.

For the BiFC assay, the CDSs of the *TaD14-4D* and *4D-HapII* were fused with N-terminal YFP, and *TaMAX2* and *TaD53* were fused with C-terminal YFP.

Different combinations of the above recombinant constructs were coinfiltrated into *Nicotiana benthamiana* leaves by *Agrobacterium tumefaciens* mediated transformation. The corresponding empty vectors were used as negative controls. The LUC and YFP signals were observed 48 72 h after infiltration by the Night SHADE LB 985 Plant Imaging System (Berthold Technologies, Bad Wildbad, Germany) and confocal microscope (LSM880; Carl Zeiss, Oberkochen, Germany).

### 4.8. SNP Detection and Molecular Marker Development

Natural variations in the presence of *TaD14* genes were detected in 32 common wheat cultivars (Table S2) with high genetic diversity. The genomic and the promoter sequences of *TaD14* genes were amplified from 32 common wheat cultivars by PCR. After sequencing, the sequences were aligned by DNAMAN software to identify polymorphism.

Two molecular markers were developed based on polymorphism sites of *TaD14-4D*. Named *dCAPS-250* and *Indel-747*, these markers were used to distinguish the three haplotypes of *TaD14-4D*. According to the difference in polymorphism, these molecular markers were classified into two types: dCAPS marker and Indel marker. The dCAPS marker was developed by dCAPS Finder 2.0 (http://helix.wustl.edu/dcaps/dcaps.html (accessed on 23 July 2020)). Genotyping was performed by two rounds of PCR and one enzyme digestion. In the first round, the genome- or promoter-specific primer was used to amplify fragments, and the reaction system was the same as gene cloning. Then, the PCR product was diluted 10 times, and 1 μL was taken as a template for the second round of PCR with dCAPS primers. The second round of PCR was performed in a volume of 10 μL, which contained 5 μL 2× Es Taq Master Mix (CWBIO), 3.5 μL ddH_2_O, 0.5 μL primer, and 1 μL template. After the two-step PCR and enzyme digestion, the amplified product containing the restriction enzyme site could be cleaved by the corresponding enzyme. However, the Indel marker required only two rounds of PCR. Finally, the fragments were separated by electrophoresis in 4% agarose gel.

### 4.9. Statistical Analysis

One-way analysis of variance was performed using IBM SPSS Statistics for Windows version 20.0 (IBM, Armonk, NY, USA) to determine the significance of differences in phenotypic traits among the three haplotypes.

## Figures and Tables

**Table 1:** *TaD14-4D* haplotypes associated with agronomic traits in MCC in four environments.

Environment	Haplotype	PH (cm)	ETN	TKW (g)
2002LY	*4D-HapI*	108.10±1.35a	7.93±0.19ab	37.54±0.54a
	*4D-HapII*	107.50±4.67a	9.38±0.80a	33.14±1.32b
	*4D-HapIII*	107.96±4.33a	6.82±0.71b	39.07±1.40a
2005LY	*4D-HapI*	103.93±1.04a	8.91±0.21a	34.86±0.46ab
	*4D-HapII*	104.18±5.84a	10.98±1.25b	31.19±1.19a
	*4D-HapIII*	105.72±3.34a	8.43±0.58a	36.48±1.20b
2006XX	*4D-HapI*	116.35±1.21a	9.24±0.19a	36.08±0.42a
	*4D-HapII*	114.90±6.28a	11.13±0.58b	32.05±1.12b
	*4D-HapIII*	112.27±3.68a	8.66±0.72a	37.79±1.28a
2010SY	*4D-HapI*	103.98±1.10a	12.60±0.24a(AB)	35.73±0.44A
	*4D-HapII*	102.72±5.19a	14.74±1.14b(A)	28.85±1.26B
	*4D-HapIII*	102.05±3.01a	11.66±0.84a(B)	38.26±1.36A

PH, plant height; ETN, effective tiller number; TKW, thousand kernel weight; 2002 LY, Luoyang (2002); 2005 LY, Luoyang (2005); 2006 XX, Xinxiang (2006); 2010 SY, Shunyi (2010). Uppercase letters and lowercase letters indicate extremely significant (*p* < 0.01) and significant differences (*p* < 0.05) between haplotypes, respectively.

## Supplementary Materials

The following are available online at https://www.mdpi.com/article/10.3390/ijms22073748/s1: Figure S1: Protein structures of TaD14-4A, TaD14-4B and TaD14-4D. Figure S2: Subcellular localization of 4D-HapII, TaMAX2 and TaD53 in wheat leaf protoplasts. Table S1: Primers used in this study. Table S2: The 32 wheat accessions used for polymorphism discovery.
